# A revamped MIC-McKeown operation without removing azygos vein arch, bronchial artery and vagus nerve trunk

**DOI:** 10.1186/s12893-023-01903-0

**Published:** 2023-03-18

**Authors:** Hai Zhang, Ying Chen, Bomeng Wu, Ying Chen, Haiquan He, Lanjuan Gong, Linrong Zhou, Cui Li, Jing Xie, Wanli Lin

**Affiliations:** Department of Thoracic Surgery, Gaozhou People’s Hospital, Affiliated to Guangdong Medical University, Guangdong Esophageal Cancer Institute Gaozhou Branch, 89 Xiguan Road, Gaozhou, 525200 Guangdong People’s Republic of China

**Keywords:** Esophageal malignant tumor, McKeown surgery, Vagus nerve, Gastrointestinal function

## Abstract

**Background:**

The purpose of this study was to investigate the effect of our revamped MIE-McKeown operation on postoperative gastrointestinal function recovery.

**Methods:**

This revamped MIE-McKeown operation without removing azygos vein arch, bronchial artery and vagus nerve trunk and with the tubular stomach buried throughout esophageal bed and azygos arch, has been implemented from July 2020 to July 2021 by the same medical team of Gaozhou People's Hospital thoracic surgery for 13 times. Preoperative clinical data, main intraoperative indicators and postoperative complications were observed.

**Results:**

All patients had esophageal malignant tumors at the level of middle and lower thoracic non-azygous venous arch, with preoperative clinical stage CT1-2N0M0 stage i-ii. V-vst test was performed on the 7th postoperative day, and 10 patients were found to have no loss of safety/efficacy. There were 2 cases with impaired efficacy and no impaired safety, 1 case with impaired safety. There were 1 cases of pulmonary infection, 1 cases of anastomotic fistula combined with pleural and gastric fistula, 2 cases of hoarseness, 2 cases of arrhythmia, 10 cases of swallowing function were grade i, 2 cases of swallowing function were grade iii, 1 case of swallowing function was grade iv in watian drinking water test one month after operation.

**Conclusions:**

Merit of this revamped MIE-McKeown operation is well preserving the integrity of azygos arch of vagus nerve and bronchial artery, and it is technically safe and feasible. No postoperative mechanical obstruction of thoracostomach, huge thoracostomach and gastrointestinal dysfunction occurs.

## Background

Radical resection of esophageal cancer is the preferred method for early and middle esophageal cancer. Traditional radical surgery for esophageal cancer is mostly anatomic esophagectomy, systematic lymph node dissection, and tubular gastroesophageal anastomosis. Those operations bring about large surgical trauma, involving a wide area of functional damage. Clinically, sequelae like huge thoracic, gastric and gastrointestinal dysfunction are prevalent, affecting postoperative rehabilitation and quality of life of patients [1, 2]. With the deepening of the understanding of esophageal cancer, the continuous innovation of minimally invasive surgical techniques for esophageal cancer, the improvement of medical instruments, and the clinical application of energy and high-definition video-assisted thoracoscopy and other working platforms, minimally invasive surgical treatment of esophageal cancer has been continuously promoted, gradually changing from the original anatomical surgical approach to the functional surgical approach [3]. Under the premise of not violating the principle of radical tumor treatment, it is the common interest pursuit of clinical workers and patients to protect the function of the non-target resection area around the esophagus more, thus improving the quality of life of postoperative patients [4]. After many attempts by previous clinical workers, it is found that the preservation of bronchial artery and vagus nerve trunk often leads to good results, but there are few reports on preservation of azygos vein arch, bronchial artery and vagus nerve trunk [5, 6]. This article summarized the merits of revamped mic-Mckeown operation and explored the safety, feasibility and effectiveness of this operation in clinical practice.

## Materials and methods

### The subjects of study

Clinical data were obtained from 13 cases of esophageal cancer patients underwent the revamped Mic-McKeown surgery performed by the same medical team in the Department of Thoracic Surgery at Gaozhou People's Hospital between July 2020 and July 2021. Group criteria: A. Patients who were newly treated and confirmed by electronic gastroscopy and biopsy pathology as thoracic esophageal squamous cell carcinoma; B. The patients were diagnosed through the ultrasound gastroscopy combined with chest and upper abdominal enhanced CT, and their multidisciplinary evaluations were almost CT1-2N0M0, with the TNM staging stage I–II (eighth edition AJCC) [7], and all their tumors were middle and lower thoracic esophageal malignancies, instead of the tumors at azygous vein arch; C. No chronic and uncontrolled diseases or serious comorbidities; D. With fine cardiopulmonary function and the tolerance to general anesthesia. This project was authorized and approved by Gaozhou Medical Ethics Committee on November 12, 2019, certificate No.: GYLLPJ-2019064.

### The main steps of minimally invasive surgery

Single lumen endotracheal intubation was performed under general anesthesia. The patient was placed in left decubitus position, and the artificial pneumothorax was established. Revamped Mic-McKeown was performed by examining external invasion of esophageal cancer and regional lymph node enlargement and relationship to surrounding tissue to determine that the vagus nerve trunk and its lower thoracic esophageal plexus were not under tumor attack.

**Fig. 1:**
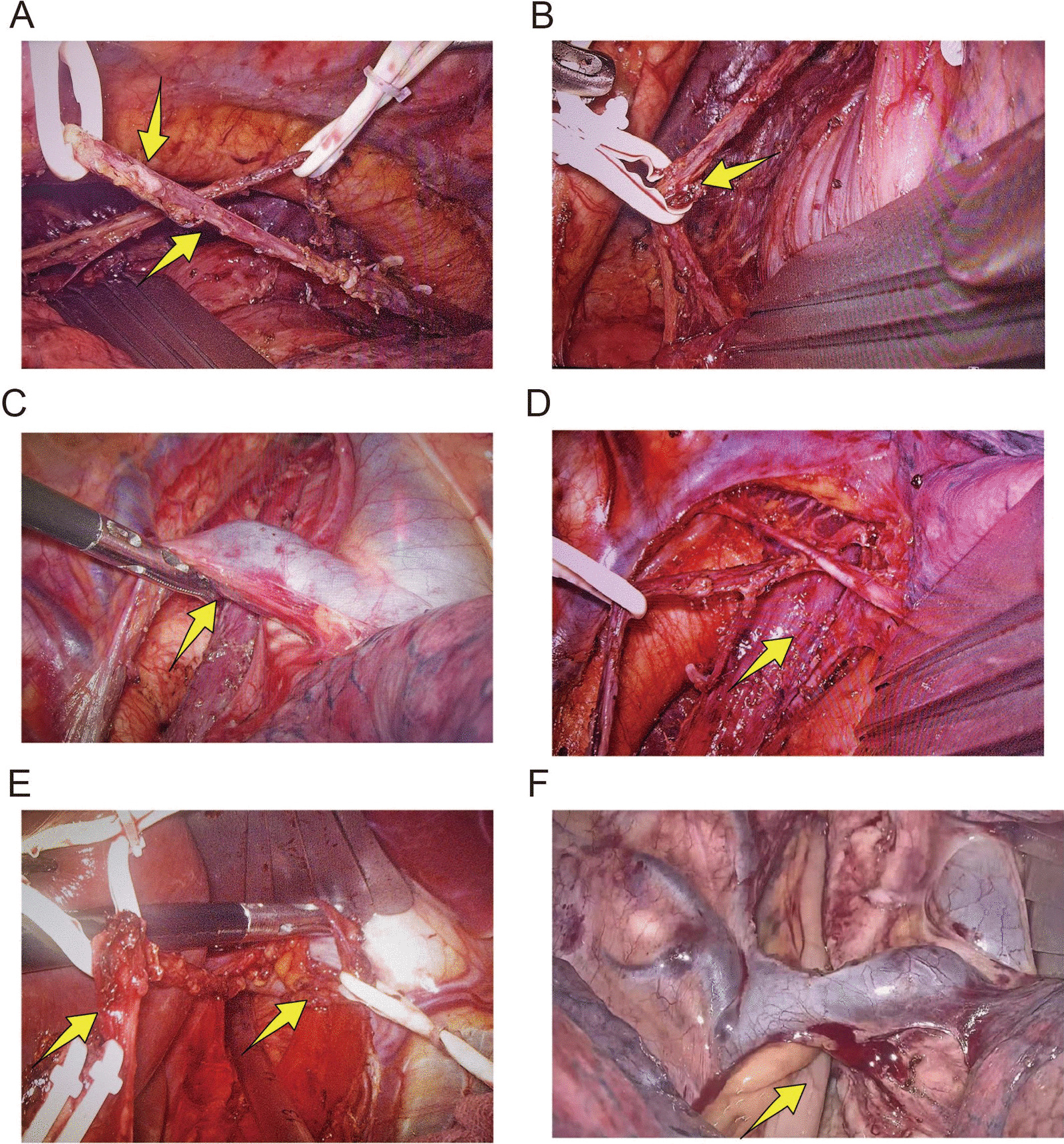
Main operating steps of minimally invasive surgery. **A** Anterior and posterior trunk of the vagus nerve. **B** The vagus plexus of a segmental esophagus. **C** Fully dissociated azygos arch and arch structure after lymph node dissection. **D** Preserved azygos vein arch, thoracic duct, right bronchial artery, right vagus trunk, and carina of lymph node after dissection. **E** hepatic and celiac branches of vagus nerve. **F** A tubular stomach was embedded through the esophageal bed and under the azygos arch

The right vagus nerve trunk of the superior mediastinum and the right recurrent laryngeal nerve were fully exposed, and the lymph nodes and adipogenic tissue of the 4R group were dissected to dissociate the upper thoracic esophagus and the lymph nodes of the 8U group; along with the azygos vein arch, the mediastinal pleura located in the lower part of the chest is opened (Fig. 1A); Azygos vein arch and right subarcuate bronchial artery should be adequately protected (Fig. 1B); the hilum of the lung was pulled forward, the esophagus was dissociated along one side of the hilum, and the right vagus nerve trunk was exposed and suspended by an undamaged strip for easy pulling and dissection. The pulmonary branch of the vagus nerve from the carina level to the lower pulmonary vein level towards the hilum should be preserved as far as possible. The anterior thoracic aorta esophagus was separated and repositioned forward. The left vagus nerve trunk was exposed above the left main bronchus between the esophagus and the aorta under azygos arch, and then suspended by a strip without injury. The bilateral vagus nerve trunks were dissociated to the esophageal hiatus (Fig. 1C), the main vagus nerve trunks were retained (Fig. 1D), and the small branches were severed. The left recurrent laryngeal nerve was identified for dissection of surrounding lymph nodes and adipose tissue.Thoroughly hemostasis was performed on the thorax, and bilateral strips were placed on the diaphragm before chest closure.Lymph nodes of 2L, 4L, 7, 8 M, 8Lo, 9R and 15 groups were sequentially dissected.

The patient was transferred to the supine position, artificial pneumoperitoneum was established, 5-hole laparoscopic surgery was performed, the gastric body was lifted, the gastric diaphragm, spleen and stomach, liver and stomach, gastrocolic and gastropancreatic ligaments were separated, clamped and cut off, the left and right gastric arteries were cut by ultrasonic knife, and the broken ends were clamped double. The right gastroomental artery was retained in the greater curvature of the stomach and the gastric body was dissociated to the pyloric ring. Firstly, the anterior trunk of the vagus nerve was lifted up and separated from top to bottom to find the liver branch and protect it. Then, the stomach was lifted up and the posterior trunk of the vagus nerve was dissociated to find the abdominal branch (Fig. 1E) and protect it. The lymph nodes of groups 16, 17, 18 and 19 were removed. A 4 cm incision was made in the middle of the epigastrium. An incision of about 5 cm in length was made inside the sternocleidomastoid muscle of the left neck, and the esophagus was lifted and pulled out from the incision in the left neck. A purse-string suture with no. 7 silk thread running through the whole layer of the esophageal wall was made around the esophagus 5 cm above the esophageal tumor, and the esophagus was cut at 0.5 cm below the suture line, and the broken end of the esophagus was ligated. The fundus of the stomach was lifted through an incision in the upper abdomen, and the stomach body and esophagus were lifted outside the abdominal wall. The stomach body was closed and cut off at 2 cm outside the cardia and lesser curvature of the stomach with a stapler, and the stomach body was cut into a tubular stomach with a stapler, the cut end of the stomach was then continuously sutured and reinforced with 3–0 sliding wire. Three pint-shaped sutures were made in the stomach fundus, and the sutures were connected with the sutures of the upper end of the ligation esophagus, and then pulled to the neck through the esophageal bed and under the azygos arch (Fig. 1F).

The tubular stomach was pulled out from the left neck incision and anastomosed with a suitable circular stapler according to the size of the broken end of the esophagus.

### Obvervational indexes

Preoperative clinical data, main intraoperative indicators and postoperative complications; Digestive tract angiography of the maximum transverse diameter of thoracic stomach on the 7th day after surgery, v-VST test results on the 7th day after surgery, and swallowing function evaluation on the 1st month after surgery (30-ml water swallowing test in Weitian Drinking water Test).

V-VST test [8]: A no loss of safety/efficacy; B With impaired effectiveness, not impaired safety; C With impaired safety (with/without impaired effectiveness).

Evaluation of drinking water test results in the depression field [9]: There are five levels.

level I, water can be drunk in one time, within 5 s, no choking; level II: requires more than 2 swallowing to finish the drink, more than 5 s, but is not accompanied by hoarseness or coughing; level III: only one swallow can swallow all the water, but accompanied by hoarseness or cough; level IV: need to swallow more than 2 times to drink water, accompanied by hoarseness or coughing; level V, persistent cough during swallowing, difficulty in drinking 30 mL water completely.

## Results

Two of the 13 patients were male, with an average age of 70.92 ± 7.70 years; Clinical stage I: 6 cases, stage II: 7 cases, middle esophagus: 12 cases, lower esophagus: 1 case. Preoperative clinical data of patients were detailed in Table 1.

**Table 1:** Preoperative clinical data of patients

Clinical features	Gender	Age (y)	T stage	Pathological stage	Tumor location	Tumor length (mm)
Case1	Female	55	T1	I	Middle	10
Case2	Female	71	T1	I	Middle	35
Case3	Female	80	T2	II	Middle	30
Case4	Male	67	T2	II	Middle	50
Case5	Female	70	T1	I	Middle	20
Case6	Female	76	T2	II	Middle	20
Case7	Male	78	T2	II	Middle	10
Case8	Female	81	T1	I	Middle	10
Case9	Female	65	T1	I	Middle	50
Case10	Female	75	T1	I	Middle	29
Case11	Female	66	T2	II	Middle	40
Case12	Female	62	T2	II	Lower	50
Case13	Female	76	T2	II	Middle	30

All the 13 patients successfully completed operation. The number of intraoperative lymph node dissection, operation time, blood loss, postoperative thoracic fluid drainage, postoperative ICU stay time, postoperative anal exhaust, postoperative feeding time, postoperative pathological stages and complications were shown in Table 2.

**Table 2:** Intraoperative and postoperative clinical data of patients

Clinical features	Case (n = 13)
Number of lymph nodes dissected	27.38 ± 12.37
Operation time (min)	297.69 ± 38.82
Intraoperative blood loss (ml)	65.38 ± 21.45
Thoracic drainage volume within 3 days after surgery (ml)	640.77 ± 347.77
Total thoracic drainage volume (ml)	959.23 ± 732.42
Duration of thoracic drainage (d)	5.46 ± 3.38
ICU stay (h)	22.25 ± 12.24
Postoperative anal exhaust(d)	3.15 ± 0.55
Postoperative fasting time (d)	7.15 ± 1.57
Postoperative hospital stay (d)	12.00 ± 3.06
G stage (%)	
G1	7.69
G2	61.54
G3	30.77
T stage (%)	
T1	53.85
T2	23.08
T3	23.07
N stage (%)	
N0	61.54
N1	23.08
N2	15.38
Pathological stage (%)	
I	38.46
II	23.08
III	38.46
Postoperative complications (%)	
Anastomotic fistula	7.69
Pulmonary infection	7.69
Cardiac arrhythmia	15.38
Hoarseness	15.38

Results of the maximum diameter of chest and stomach, V-VT test, gastrointestinal symptoms and 30 ml water test 1 month after the operation of 13 patients on the 7th day after the operation were shown in Table 3.

**Table 3:** Clinical data of the patients on the maximum diameter of the thoracic cavity and stomach, V-VT test, gastrointestinal symptoms and 30 ml water drinking test at 1 month postoperatively

Clinical features	Case (n = 13)
The maximum diameter of chest and stomach on the 7th day after surgery(mm)	20.54 ± 5.85
V-vt test was performed on the 7th postoperative day	
A (cases)	10
B (cases)	2
C (cases)	1
Gastrointestinal symptoms one month after operation	
Stomachache (cases)	0
Ventosity (cases)	1
Diarrhea (cases)	1
STOMACH o (cases)	9
The drinking water test in the depression field one month after the operation	
I level (cases)	10
II level (cases)	0
III level (cases)	2
IV level (cases)	1
V level (cases)	0

## Discussion

The prerequisite of functional surgery for esophageal cancer is not to put away the tumor R0 rate and to violate the principle of radical treatment [10, 11]. In lights of this condition, all the cases mentioned in this paper were selected as ct1-2N0M0 malignant tumors of the lower and middle thoracic segment at the level of azygos venous arch in clinical stages. There were abundant reticular lymphatic vessels in the submucosa of the esophagus. With the escalation of tumor T stage, the infiltration depth deepened, and the reticular lymphatic network was involved. Tumor cells metastasized through the lymphatic network, and the most frequent site of metastasis was the para-laryngeal nerve region [12, 13]. Therefore, enhanced chest CT scan combined with ultrasound gastroscopy was required for all the enrolled patients, and the tumor was assessed to be confined to T2 and located in the lower and middle thoracic segment without regional lymph node metastasis. Among the 13 patients enrolled in this study, postoperative pathology confirmed negative margins, among which 5 patients showed POSTOPERATIVE N + , and adjuvant chemotherapy or chemoradiotherapy was recommended for all patients [14].

If the vagus nerve trunk and its main branches were to be preserved, it is necessary for the operating personnel to get familiar with the thoracoabdominal anatomy of the vagus nerve trunk and its main branches: The left vagus nerve continues to the anterior vagus trunk in the lower esophagus, and divides into the anterior gastric and hepatic branches near the cardia. The right vagus nerve forms the posterior trunk of the vagus nerve at the rear of the lower esophagus, and gives out the posterior gastric branch near the cardia. It goes along the back of the lesser curvature of the stomach, and branches distribute in the posterior wall of the stomach. The abdominal branch of the vagus nerve is the final branch of the posterior trunk of the vagus nerve. It is lasted until right to the vicinity of the abdominal trunk and mixes with the sympathetic nerve to form the abdominal plexus. Along with the blood vessels, it distributes in the abdominal digestive tract above the left curvature of the liver, bile, pancreas, spleen, kidney and colon [15, 16]. The vagus nerve trunk and lower thoracic esophageal plexus, abdominal and liver branches are usually retained. On the basis of the principle of radical surgical treatment and duo to the limitations of surgical techniques, only the thicker branches of anterior and posterior vagus plexus are the only sections could be reserved. In this group, only the right vagus nerve trunk was successfully preserved in 2 cases, and the liver branch was injured in 3 cases, which was related to the unskilled operation and large variation of neuroanatomy. In thoracic surgery, it is suggested to dissect the bilateral vagus nerve trunk under the azygos vein arch and then dissociate it downward after being marked with non-invasive strips, so as to better protect the integrity of the main vagus nerve. In abdominal surgery, it is recommended to open the peritoneum, find the mark of vagus nerve skin that was originally placed in the chest, and then pull it into the abdominal cavity before dissociating the stomach, so as to better expose and protect the liver and abdominal branches.

**Fig. 2:**
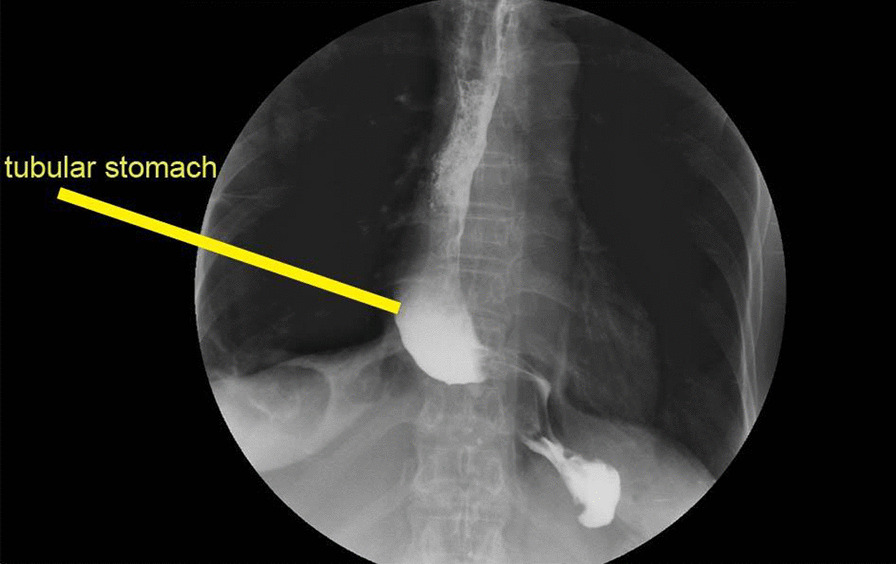
X-ray findings of upper gastrointestinal angiography on the 7th postoperative day

Minimally invasive McKeown and tubular stomach are the conventional means for digestive tract reconstruction. Postoperative thoracic stomach will impinge on cardiopulmonary function and gastrointestinal dysfunction, and reduce the life quality of patients. In previous studies, in order to reduce this complication, different studies have discussed the placement of the tubular stomach (retrosternal path, trans-mediastinal esophageal para-bed path) and the size of the tubular stomach, with different results [17, 18]. In this study, the azygos venous arch was retained through the mediastinal esophagus beside the bed. Mechanically, the two ends of azygos venous arch were equivalent to two sets of screw fixation principle. The arch was a cantilever beam structure, and the tubular stomach was embedded under the azygos venous arch of the esophagus bed, which was equivalent to two sets of screws fixing the cantilever beam structure to fix the tubular stomach in the esophagus bed; while the azygous vein arch is fully free and itself is a soft lumen structure, which expands during feeding in the tubular stomach to avoid mechanical obstruction, as shown in Fig. 2. On the 7th day after operation, the tubular stomach was fixed in the esophageal bed without obvious chest and stomach dilation, which met the physiological requirements.

Gastrointestinal dysfunction is a common postoperative complication of esophageal cancer. At present, it is thought that gastrointestinal dysfunction is related to vagus nerve injury and function limitation, or early sympathetic nerve excitation. After vagus nerve trunk was cut off, the small intestine to promote power hormone secretion declined, at the same time gastric peristalsis lost inhibiting ectopic pacemaker, making the gastric antrum and duodenum peristaltic wave separation pressure, continuity of the gastrointestinal peristalsis wave disturbance, stomach food retention phase extension and emptying delay, cause gastric emptying disorder, or pyloric sphincter is out of control, and cause emptying too fast, cause diarrhea [19]. Among the 13 patients involved in the study, abdominal distention and diarrhea occurred in 1 case within 1 month after surgery, and the symptoms did not need drug intervention, reflecting the preservation of vagus nerve, which plays a promoting role in postoperative gastrointestinal function recovery and can better prevent gastrointestinal dysfunction.

## Conclusion

Under revamped Mic-McKeown operation, the bronchial artery, the main vagus nerve trunk, has been preserved; moreover, under the premise of ensuring the principle of oncology treatment, the operation time and blood loss were not increased, the integrity of azygos venous arch of vagus nerve and bronchial artery was better preserved, and postoperative mechanical obstruction of pleural stomach, huge pleural stomach and gastrointestinal dysfunction was not occurred, which was more in line with physiological requirements.

## Data Availability

The datasets used and/or analysed during the current study are available from the corresponding author on reasonable request.
